# Electric field modulated redox-driven protonation and hydration energetics in energy converting enzymes†

**DOI:** 10.1039/c9cc01135h

**Published:** 2019-05-23

**Authors:** Patricia Saura, Daniel M. Frey, Ana P. Gamiz-Hernandez, Ville R. I. Kaila

**Affiliations:** Center for Integrated Protein Science Munich (CIPSM), Department Chemie, Technische Universität München, Lichtenbergstraße 4, 85748, Garching, Germany

## Abstract

Biological energy conversion is catalysed by proton-coupled electron transfer (PCET) reactions that form the chemical basis of respiratory and photosynthetic enzymes. Despite recent advances in structural, biophysical, and computational experiments, the mechanistic principles of these reactions still remain elusive. Based on common functional features observed in redox enzymes, we study here generic mechanistic models for water-mediated long-range PCET reactions. We show how a redox reaction within a buried protein environment creates an electric field that induces hydration changes between the proton acceptor and donor groups, and in turn, lowers the reaction barrier and increases the thermodynamic driving forces for the water-mediated PCET process. We predict linear free energy relationships, and discuss the proposed mechanism in context of PCET in cytochrome *c* oxidase.

Biological energy conversion is powered by membrane-bound enzymes that convert chemical and light-energy into an electrochemical ion gradient, stored across a biological membrane.^1^ These enzymes form the biochemical basis of cell respiration or photosynthesis, and they provide a molecular basis for cells to harness energy and to power energy-requiring processes. Interestingly, the main energy transducing enzymes in nature are powered by elementary proton-coupled electron transfer (PCET) reactions, which involve stepwise or concerted transfer of protons (H^+^) and electrons (e^−^), between the same or different donor/acceptor groups.^2–6^ The recent structural revolution has provided atomic-scale blueprints of these systems,^7–9^ which together with biophysical and computational experiments, provide a chemical basis for elucidating mechanistic principles of PCET reactions in biology.

In contrast to hydrogen atom or hydride transfer reactions that take place within chemical bonding distances,^2–6^ many biological systems catalyse long-range PCET reactions, which involve >10 Å charge transfer separation reactions that can be kinetically limited by either the redox or the protonation reaction. Since protons do not tunnel across such large distances, water molecules form an intricate part of such PCET-systems by providing conduits that enable the proton transfer (pT) reaction. Hydration and dehydration processes can therefore modulate the kinetic barriers for such long-range PCET reactions.

Long-range PCET is often initiated by a redox process in the enzyme’s active site, which creates an electric field. To minimise the overall energy of the system, water molecules move into this non-uniform electric field, and form water arrays, opposing the initial redox-field. The same electric forces can also mobilise protons that travel along the water molecules to the redox site or its vicinity, by a Grotthuss mechanism,^10^ where the charge rather than the proton itself diffuses along the water chain, followed by re-orientation of the dipole direction. Such redox-linked hydration and protonation changes have been observed in several enzymes: in cytochrome *c* oxidase (C*c*O), which functions as a terminal electron acceptor in aerobic respiratory chains, where the reduction of its active site directs protons both across the membrane and to the active site.^11–14^ In respiratory complex I, also a redox-driven proton pump in respiratory chains, a similar field-effect induced by quinone reduction in the active site, leads to local protonation and conformational changes that propagate across the *ca.* 200 Å wide membrane domain of the enzyme.^15–19^ Moreover, electric-field-induced effects may also play a role in water splitting of photosystem II,^20^ where the stepwise oxidation of the water-splitting Mn_4_O_5_Ca site directs protons across the membrane. However, despite these mechanistic insights, the exact chemical basis for how the long-range PCET reactions are gated by hydration and protonation reactions still remains unclear.

Here we study the energetics of water-mediated PCET reactions based on generic functional elements found in many energy-converting enzymes. Despite its simplicity, the model captures key features of long-range charge transport effects in biology and predicts how kinetic gating effects could be achieved on a molecular level. Mechanistic principles of the model are further tested on PCET reactions in C*c*O.

To probe the coupling between the protonation and hydration energetics, and the redox chemistry, we created a model system comprising a redox-controlled proton acceptor (A), which is separated by a quasi-one-dimensional water array from a proton donor (D) (Fig. 1 and Fig. S1, ESI†). The model mimics the interior of a buried active site in membrane-bound PCET-enzymes and the dimensions of proton channels in, *e.g.*, C*c*O and complex I where the charge-modulating groups are separated by *ca.* 15 Å from the bulk.^12,16–19^

The energetics estimated using density functional theory (see ESI†) for the water-mediated pT reactions is shown in Fig. 2. Prior to the redox-reaction, the free energy barrier is *ca.* 17 kcal mol^−1^ (Δ*H*^‡^ = 18 kcal mol^−1^, ΔZPE^‡^ = −1.9 kcal mol^−1^, *T*Δ*S*^‡^ = −0.7 kcal mol^−1^, Fig. S1, Table S1, ESI† and Fig. 3) for the *n* = 3 water molecule system, placing the reaction in the seconds timescale according to transition state theory. Energetics in the DFT cluster models are similar as in our explicit lipid models, probably due to the low dielectric shielding effect of the hydrophobic membrane interior (see Fig. S1, ESI†). The results presented below thus refer to DFT cluster models. For longer (*n* = 5) water chains, the barrier decreases due to interactions of the transition state (TS) with the antiparallel water dipoles on each side of the central protonated water species, whereas for *n* = 1, the TS energy is lowered probably due to direct orbital interaction with the D/A groups. We find that when the pathway between the D and A groups is dry, the reaction barrier significantly increases to *ca.* 40 kcal mol^−1^ (Fig. S2, ESI†) suggesting that a well-connected pathway provides a pre-requisite for the proton transfer reaction by lowering the energy of the charge separated state. Perturbation of the proton acceptor side by a redox reaction (*δq* = −1 reduction; *δq* = +1 oxidation) leads to a parabolic shift of the donor state, the transition state, and the acceptor state (Fig. 1b). This perturbation arises from the interaction between the excess charge and the non-uniform electric field created by the tuneable redoxsite. A unit charge leads to an electric field change of around 0.2–0.5 V Å^−1^ (Fig. 2 and 3), which opposes the field of the water array and transferred proton.

The energy barrier and thermodynamic driving force as a function of the perturbing charge and field are shown in Fig. 2. In the studied field range, the calculations predict that both the reaction barrier and chemical driving force decrease linearly with the redox reaction. The reaction barrier is thus predicted to have a linear dependence on the chemical driving force with a Brønsted slope of 0.1–0.4 for the forward reaction, and 0.9–0.6 for the backward reaction (Fig. 3b and c), with resemblance to previously studied PCET and pT systems,^21–23^ and recent experiments.^24,25^ A similar linear energy relationship is also predicted from an analytic electrostatic treatment, with slopes approaching 1 and 0 as *n* → ∞ (Fig. S3, ESI†) *cf.* also.^21–23^ The redox reaction reduces the forward (D → A) reaction barrier in all systems by *ca.* 5 kcal mol^−1^, whereas the backward barrier (A → D) increases: for the *n* = 3 water system, the redox reaction decreases the forward barrier from 18 kcal mol^−1^ to *ca.* 13 kcal mol^−1^, which is expected to decrease the reaction rate from seconds to the milliseconds timescale based on transition state theory, whereas the backward reaction barrier increases by > 30 kcal mol^−1^, making it kinetically inaccessible. This effect could enhance the directionality of the PCET reaction and decrease charge recombination reactions. Interestingly, the electric field induced by the redox reaction increases the affinity for the water array by *ca.* 10 kcal mol^−1^ for the reactant state, suggesting that the redox reaction enhances the stability of the water array, which in turn, provides a pre-requisite for the proton transfer reaction (Fig. S2, ESI†). Moreover, in the product state, the water affinity is reduced by *ca.* 10 kcal mol^−1^ due to effective recombination of the proton and electron, that favours dehydration of the pT-array. Due to microscopic reversibility, the pT reaction also modulates the redox reaction. To probe these effects, we modelled the redox site as a TyrO^•^/TyrO^−^ redox couple at the end of the *n* = 3 array. We obtain a +180 mV (4.2 kcal mol^−1^) stabilisation of the electron in the product state relative to the reactant state that equals the lowering of the pT reaction energetics in the thermodynamic cycle (Fig. S4, ESI†).

To probe how the redox-driven hydration and protonation model applies to an enzymatic PCET reaction, we optimised the active site region of C*c*O based on the X-ray structure of the enzyme from *Bos taurus* using DFT calculations (Fig. 4).^26^ The model comprises *ca.* 270 atoms, including all first- and second-sphere protein residues surrounding the active site around 10 Å, and the water structure obtained from a 0.5 μs MD simulation.^13,27^

The model was studied prior and after reduction of the active site, in the so-called P_M_ (Fe^IV^═O^2−^ Cu^II^–OH^−^ TyrO^•^) and P_R_ (Fe^IV^═O^2−^ Cu^II^–OH^−^ TyrO^−^) states. This redox reaction couples to a pT from Glu-242 along a one-dimensional water chain to the active site along the so-called “*chemical proton pathway*”.^12^ Previous MD simulations suggest that the stability of the water chain^11–14,27^ is enhanced by reduction of the active site. The reaction energetics, shown in Fig. 4, predict that the reduction of the active site lowers the pT barrier by *ca.* 4 kcal mol^−1^, whereas the chemical driving force increases by *ca.* 6 kcal mol^−1^, following the predicted linear energy relationship (Fig. 2 and 3). We find that the redox reaction in the active site (TyrO^•^ → TyrO^−^) increases the magnitude of the electric field by *ca.* 0.2–0.5 V Å^−1^, *i.e.*, in a similar range as in our model systems. This directed field lowers in turn the reaction barrier proportionally to the field difference and the dipoles in the transition and reactant states, $\Delta E_{\text{f}}^{\text{ox}/\text{red}}\cdot \left(\mu^{\text{TS}}-\mu^{\text{R}}\right).$ Interestingly, when we add a uniform external electric field of 0.2 V Å^−1^ in the direction of the pT wire in the oxidised (P_M_) state, the reaction barrier also decreases by *ca.* 4 kcal mol^−1^, resembling the barrier in the reduced state, whereas the added field increases the exergonicity of the reaction to *ca.* 8 kcal mol^−1^, which is comparable to the energetics for the reduced model. The observed electric field variations arising from the PCET reaction are likely to have importance for gating the proton transfer reaction and preventing short-circuit reactions in C*c*O. Our combined findings thus suggest that key features of the proposed model also apply in enzymatic water-mediated PCET reactions.

In this work, we have studied the energetics and mechanism of long-range water-mediated PCET reactions based on DFT calculations. The model suggests that a redox reaction induces electric fields of 0.2–0.5 V Å^−1^ in non-polar protein environments that thermodynamically stabilises water arrays between the redox site and a proton donor, lowers the barrier for the forward proton transfer reaction, and subsequently increases the barrier for the backward charge recombination reaction. The model has similarities to electrostatic catalysis effects in enzymes.^28^ In the PCET reactions, the water array provides a pre-requisite for the proton transfer reaction, which is in addition to the stability of the water array itself, also modulated by the electric field arising from the electron transfer reaction. We further showed that the electric field varies up to 0.5 V Å^−1^ in the active site of cytochrome *c* oxidase, with important implications for gating the proton transfer reaction. The redox-driven hydration model is suggested to also apply to water-mediated PCET and charge transfer reactions in other energy transducing enzymes, *e.g.*, respiratory complex I,^15–19^ light-driven ion pumps,^29^ and photosystem II.^20^

See ESI† for detailed computational methods.

## Supplementary Material

† Electronic supplementary information (ESI) available: Additional data on analytical model, free energies, reaction profiles, and electron affinities. See DOI: 10.1039/c9cc01135h

Supplementary Information

## Figures and Tables

**Fig. 1:**
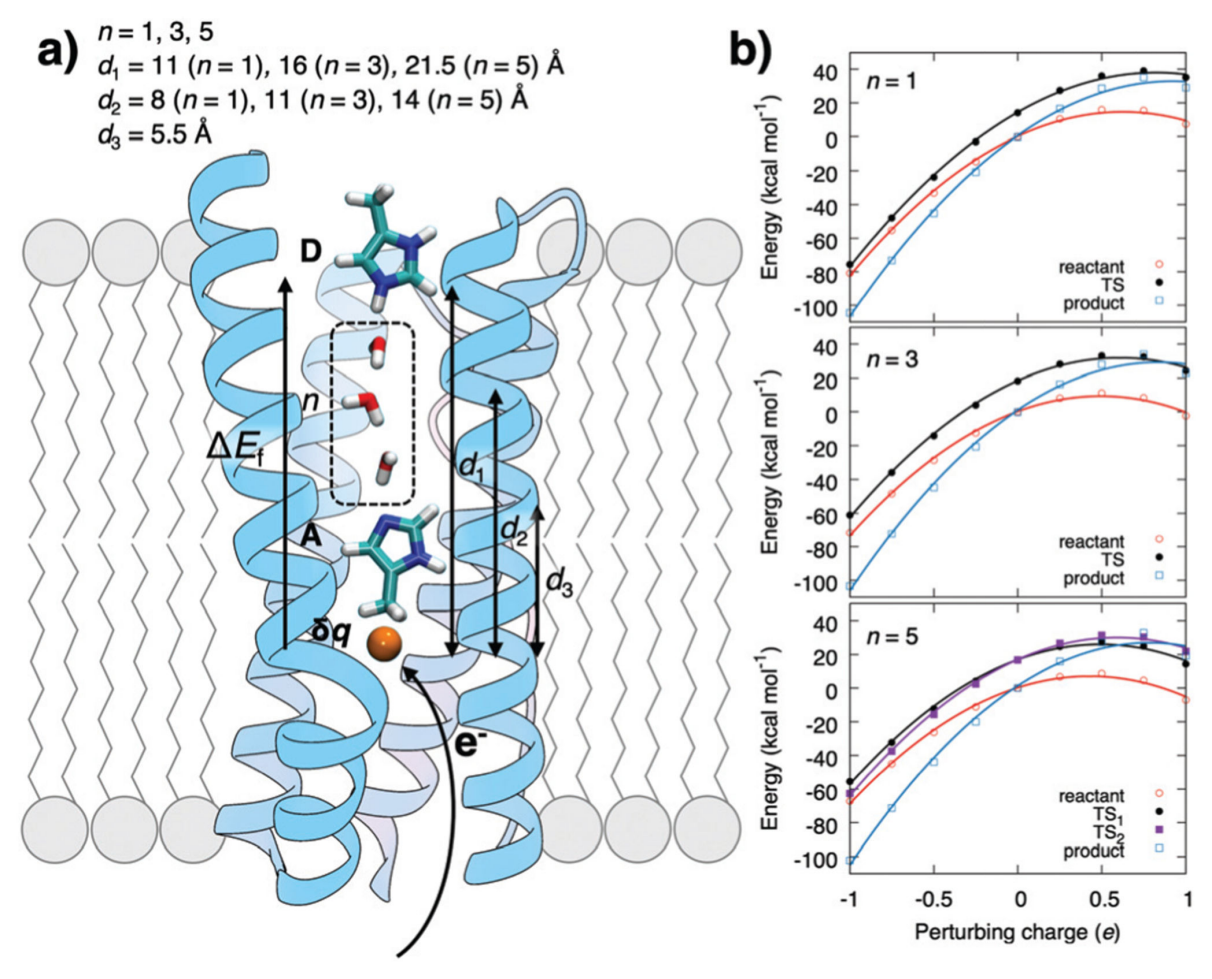
(a) Schematic model system for water-mediated PCET reactions in membrane-bound enzymes between proton donor (D) and acceptor (A) sites that are separated by a water chain composed of *n* water molecules. The redox reaction (*δq*) generates an electric field, Δ*E*_f_, that promotes the proton transfer reaction. (b) The redox reaction parabolically perturbs reactant, transition state (TS), and product state energies in DFT cluster models of the PCET reaction (see ESI† Methods).

**Fig. 2:**
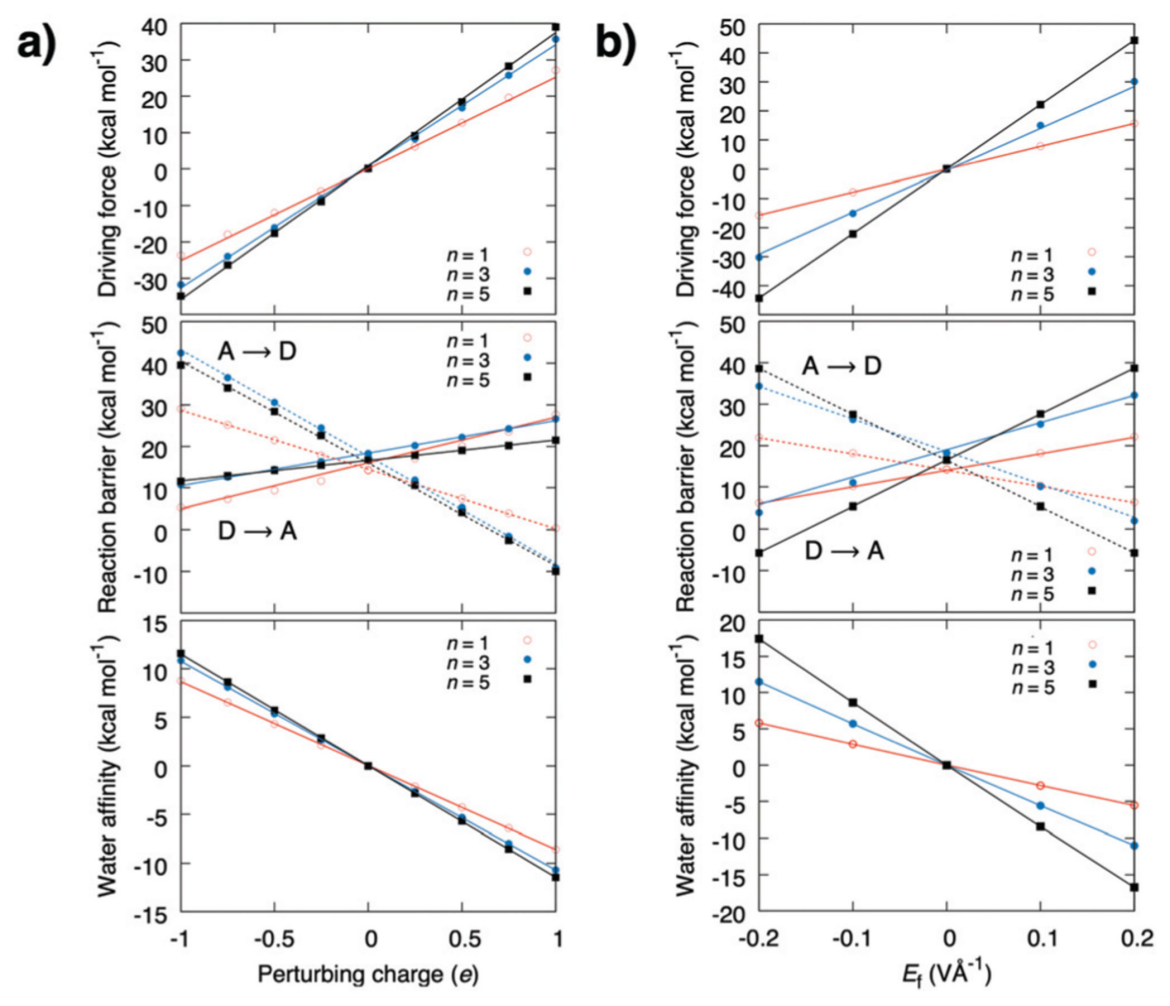
The redox reaction creates an electric field that linearly lowers the forward pT barrier, and increases the backward pT barrier and the thermo-dynamic driving force, as well as the stability of the water chain. The graphs show the driving force (top), reaction barrier (middle), and water affinity (bottom) (a) upon perturbation with *δq*, and (b) when a uniform electric field is applied along the pT direction.

**Fig. 3:**
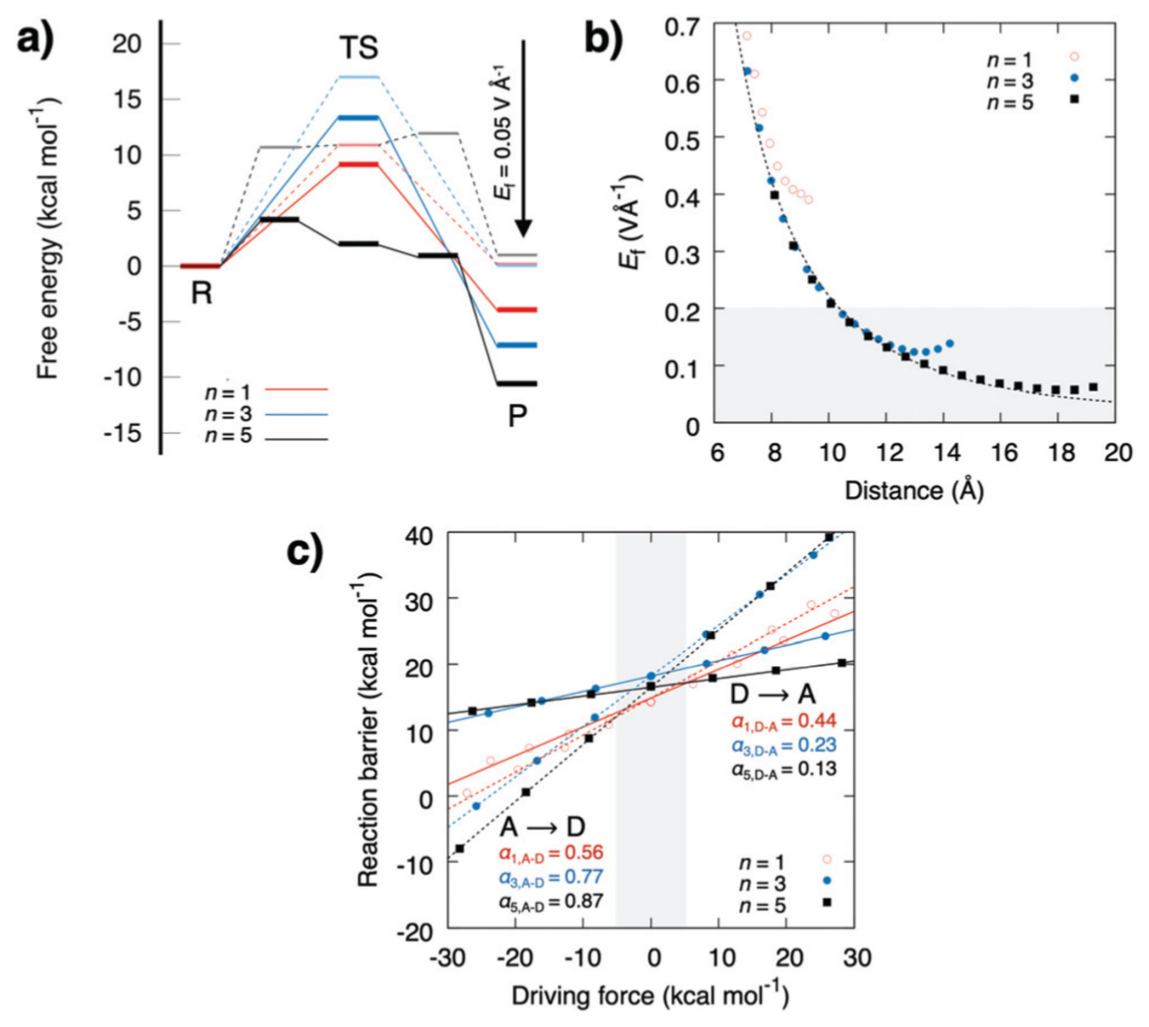
(a) Effect of pT free energy profiles upon application of a uniform 0.05 V Å^−1^ redox-field. (b) The electric field created by a redox reaction as a function of distance to the redox site, and (c) linear energy relationships between the thermodynamic driving force and the reaction barrier.

**Fig. 4:**
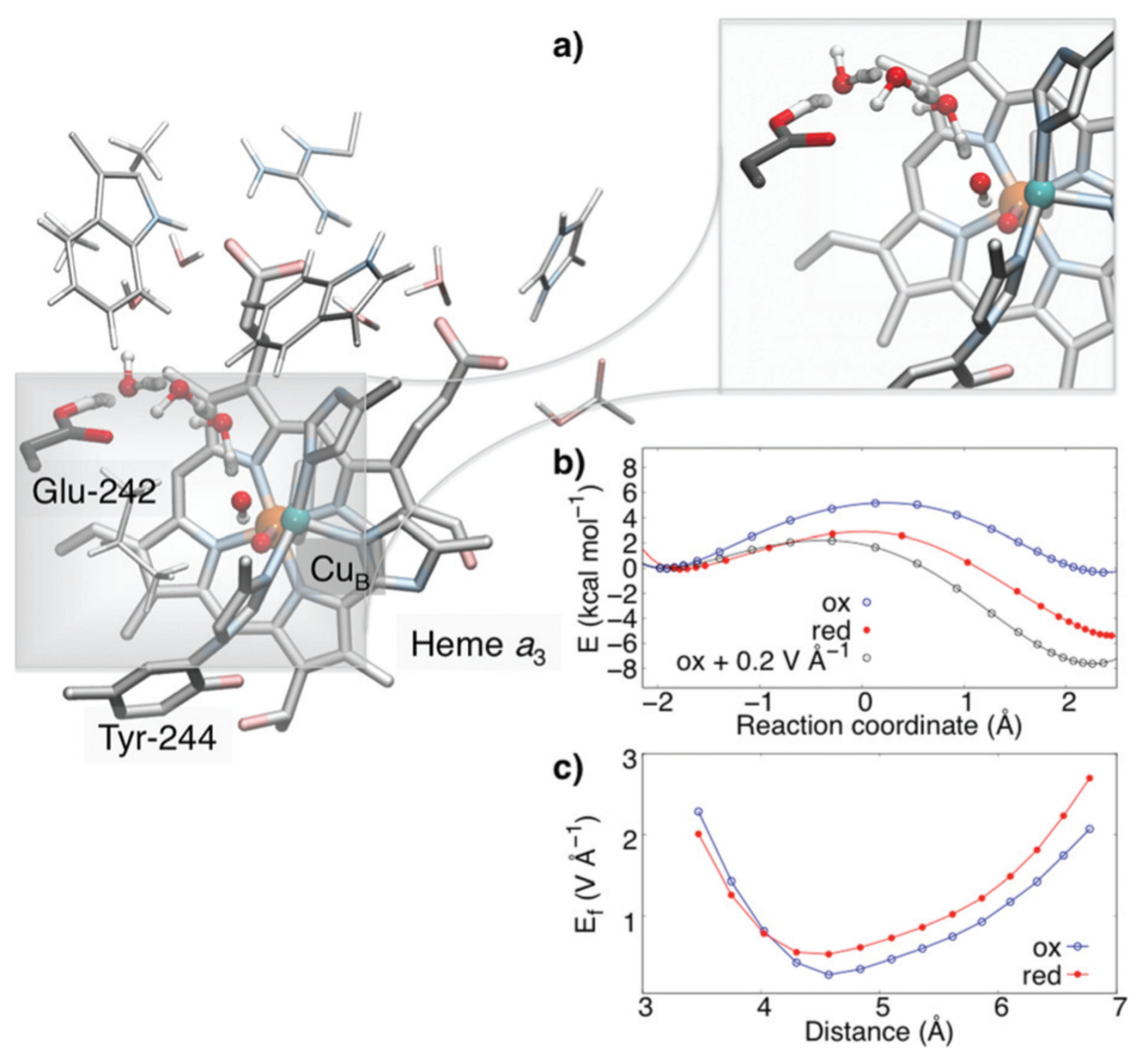
PCET in cytochrome *c* oxidase. Reduction of the heme *a*_3_/Cu_B_ leads to pT from Glu-242 *via* a water-chain to Cu_B_ (Cu^II^–OH^−^). (a) Close-up of the water-mediated pT reaction, (b) energy profiles for the pT reaction in the oxidised (P_M_) and reduced (P_R_) states, and the oxidised state upon application of an 0.2 V Å^−1^ external field aligned along the pT direction, (c) computed electric fields for the oxidised (P_M_) and reduced (P_R_) states.
