# Supplementary material for: Electric field modulated redox-driven protonation and hydration energetics in energy converting enzymes†
Source: Chem Commun (Camb). Author manuscript; Available in PMC 2019 Dec 26. (PMC6932871; doi:10.1039/c9cc01135h)
Supplement: Supplementary Information [file EMS85191-supplement-Supplementary_Information.pdf]

Electronic Supplementary Material (ESI) for ChemComm.
This journal is © The Royal Society of Chemistry 2019

Supplementary Information

Electric Field Modulated Redox-Driven Protonation and Hydration Energetics in Energy Converting Enzymes

Patricia Saura[a], Daniel Frey[a], Ana P. Gamiz-Hernandez[a], Ville R. I. Kaila*[a]

[a]Center for Integrated Protein Science Munich (CIPSM), Department Chemie, Technische Universität München, Lichtenbergstraße 4, 85748, Garching, Germany.

*Corresponding author: ville.kaila@ch.tum.de

Computational methods

DFT calculations of PCET model systems

DFT models for the studied PCET reactions comprised proton donor and acceptor groups, modelled as histidine residues, connected by a quasi-one dimensional water array with n = 1, 3 and 5 water molecules. The systems comprised 28, 34, and 40 atoms, respectively. The histidine residues were cut at the Cα-Cβ position, saturated by hydrogen atoms, and the Cβ atoms were kept fixed during geometry optimizations. The donor and acceptor groups were separated by 11, 16, and 21.5 Å. Geometry optimizations were performed at the B3LYP-D3/def2-SVP level, and single point energy calculations were performed at the B3LYP-D3/def2-TZVP level.[1–4] Reaction pathways were optimized using the *chain-of-states* method connecting reactant and product states with optimized transition states.[5] Zero-point energy (ZPE) and entropic effects were estimated at the B3LYP-D3/def2-SVP level. The redox reaction coupled to the pT-process was simulated by either including a point charge placed at a distance of 5 Å from the Nε atom of the histidine acceptor group that was tuned from $-e$ to $+e$ (Fig. 2, 3) or by modelling a $TyrO^{\bullet}/TyrO^{-}$ group placed at the same distance from the n = 3 PCET model system (main text, Fig. S4).

To probe the effect of a biological environment on the PCET reaction, the n = 3 water molecule PCET-system was embedded in a *ca.* 6 Å hole within a *ca.* 40 Å thick 1-palmitoyl-2-oleoyl-sn-glycero-3-phosphocholine (POPC) lipid membrane bilayer solvated by 20 Å layer of water molecules on both sides of the bilayer (Fig. S1) with a total dimension of *ca.* 70x70x50 $Å^3$. The effect of the lipid/water surroundings on the PCET reaction were modelled using a hybrid QM/MM approach. The total system comprised *ca.* 25,000 atoms. The MM system was represented using atomic point changes obtained from the CHARMM36 force field parameters.[6] The reaction profiles obtained in the explicit membrane model closely resembled the QM energies obtained from the DFT model with an implicit polarizable medium with a dielectric constant of ε = 2 modelled using COSMO,[7] as well as the profiles in vacuum (Fig. S1). The PCET effects were therefore explored further using the DFT models without the lipid environment (Figs. 2-3). We expect, however, that an explicit protein/membrane environment is likely to strongly perturb the PCET energetics. All QM calculations were performed with TURBOMOLE v 6.6-7.2.[8]

DFT calculations on cytochrome c oxidase

DFT models of the active site of C*c*O were built based on the crystal structure of the protein from *Bos taurus* (PDB ID: 1V54),[9] with the water structure obtained from a previous 500 ns MD simulation of the a^{ox}/P_R state.[10] The model comprised the heme a_3 and Cu_B-OH^-/H_2O cofactors, the water molecules obtained from the MD simulation, and residues Trp-126, Trp-236, Val-243, Tyr-244, Asp-364 (modelled protonated), His-368, Arg-438, His-376 coordinating heme a_3; His-240, His-290, His-291 coordinating Cu_B, and the proton donor Glu-242. His-368 was modelled in its protonated $HisH^+$ state to mimic the Mg^{2+} site. The effect of heme a was not included in the model due QM system size limitations. The QM model comprised *ca.* 270 atoms. Protein side chains were cut at the Cβ position, and was kept fixed during geometry optimizations to simulate restraints from the protein framework. Geometry optimizations were performed at the BP86-D3/def2-SVP/def2-TZVP(Fe,Cu) level[1–4] using the multi-pole accelerated resolution of identity (MARIJ-RI) and an implicit polarizable medium with dielectric constant ε=4 using a conductor-like screening model,[7] in the P_M (Fe^{IV}=O/Cu^{II}-OH^-/TyrO) and P_R (Fe^{IV}=O/Cu^{II}-OH^-/$TyrO^-$) states for the proton transfer reaction between Glu-242 and Cu_B-OH^-.

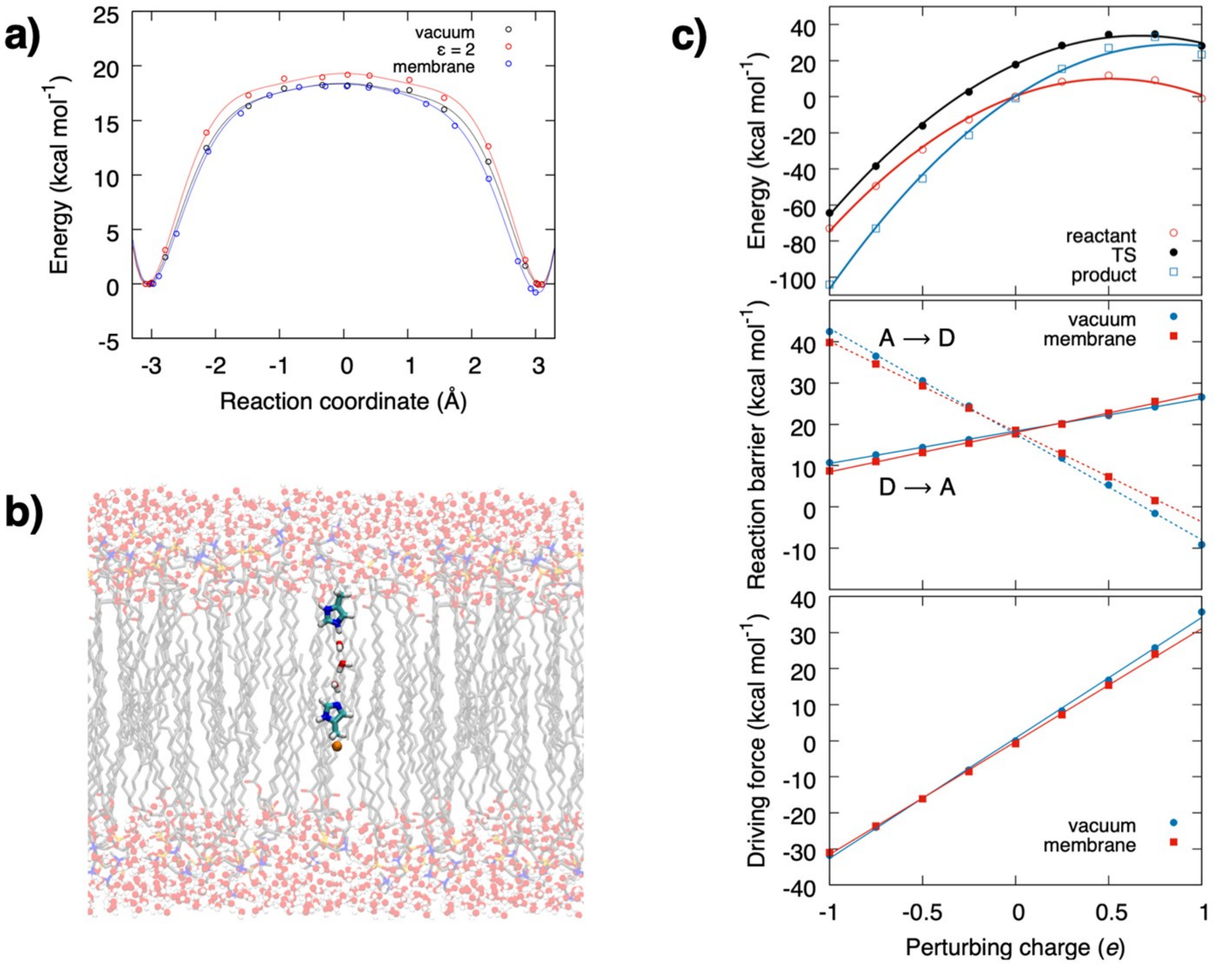

Fig. S1. a) Effect of a POPC membrane model on the PCET energetics for the $n = 3$ water model system. The reaction barriers for the proton transfer process are similar as in vacuum and $\varepsilon = 2$ models (see SI Methods). b) Structure of the model system embedded in the lipid/water environment. Hydrogen atoms of the lipid tails are not shown for clarity. c) Similar as in the DFT models, the redox reaction parabolically perturbs the reactant, transition state (TS), and product states in the membrane environment (*top*), and linearly perturbs the forward and backward reaction barriers (*middle*), as well as the thermodynamic driving force (*bottom*).

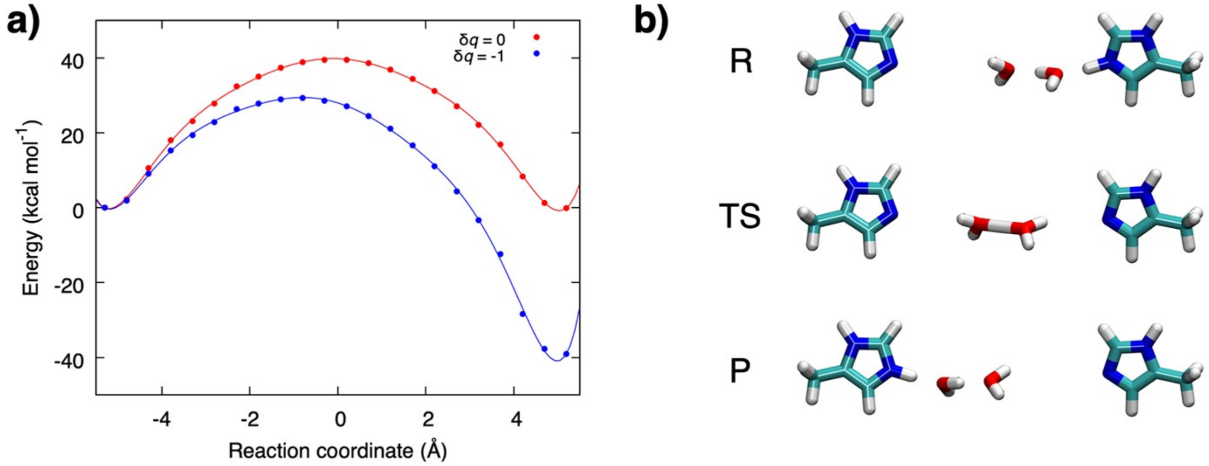

Fig. S2. a) Partial dehydration of the water array significantly increases the reaction barrier of the pT reaction 40 kcal mol^{-1} (red curve). A negative perturbing charge δ*q* = -1 (blue curve) lowers the forward D⟶A pT reaction barrier to 30 kcal mol^{-1}, and the driving force to -40 kcal mol^{-1}, whereas it increases the backward A⟶D pT reaction barrier and driving force to 70 kcal mol^{-1} and 40 kcal mol^{-1}, respectively. b) Reactant (*top*), transition state (*middle*), and product state (*bottom*) in the dehydrated model. Energies are reported at the B3LYP-D3/def2-TZVP level.

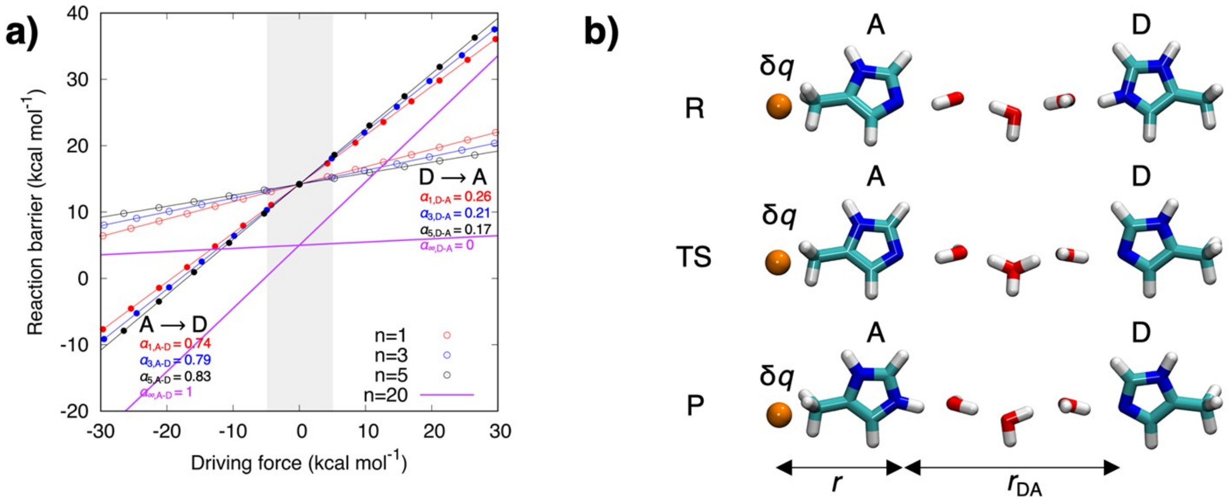

$$E^{\mathrm{D}} = \frac{\delta q}{4\pi\varepsilon\varepsilon_0}\left[\frac{-\boldsymbol{\mu}\,n}{\left(r+\frac{r_{\mathrm{DA}}}{2}\right)^2} + \frac{Q}{(r+r_{\mathrm{DA}})}\right] \tag{1}$$

$$E^{\mathrm{TS}} = \frac{\delta q}{4\pi\varepsilon\varepsilon_0}\left[\left(\frac{\boldsymbol{\mu}(n-1)}{2}\right)\left[\frac{-1}{\left(r+\frac{r_{\mathrm{DA}}}{4}\right)^2} + \frac{1}{\left(r+\frac{3r_{\mathrm{DA}}}{4}\right)^2}\right] + \frac{Q}{\left(r+\frac{r_{\mathrm{DA}}}{2}\right)}\right] \tag{2}$$

$$E^{\mathrm{A}} = \frac{\delta q}{4\pi\varepsilon\varepsilon_0}\left[\frac{+\boldsymbol{\mu}\,n}{\left(r+\frac{r_{\mathrm{DA}}}{2}\right)^2} + \frac{Q}{r}\right] \tag{3}$$

Fig. S3. Electrostatic model for the PCET reaction energetics. a) Linear energy relationship between the reaction barrier and thermodynamic driving force predicted from an analytical electrostatic model with n=1, 3, 5 water molecules, and as $n \longrightarrow \infty$. b) The model geometry with n =3 water molecules, showing the donor (D) and acceptor (A) sites, separated by a distance r_{DA}. The transition state (TS) is located at $r_{\mathrm{DA}}/2$, and the perturbing charge, δq, is placed at a distance r from A. δq generates an electric field, $\boldsymbol{E}_{\mathrm{f}}$, that orients the water dipoles ($\boldsymbol{\mu}$) with centers at (r + $r_{\mathrm{DA}}/2$) in A/D, and, (r + $r_{\mathrm{DA}}/2$) and (r + $r_{\mathrm{DA}}3/4$) in TS, relative the perturbing charge. The transferred proton, with a charge Q, is located at (r + r_{DA}) from δq in D, at (r + $r_{\mathrm{DA}}/2$) in the TS, and at r in the A state. *Bottom*: analytical expressions of the energy of the reactant state E^{D} (Eqn. 1), transition state E^{TS} (Eqn. 2) and product state E^{A} (Eqn. 3). The reaction barrier and driving force for the forward proton transfer are calculated as E^{TS}- E^{D} and E^{A}- E^{D}, and for the backward proton transfer as E^{TS}- E^{A} and E^{D}- E^{A}, respectively, with the internal chemical energy of the TS relative to the D obtained from the DFT calculations (Table S1).

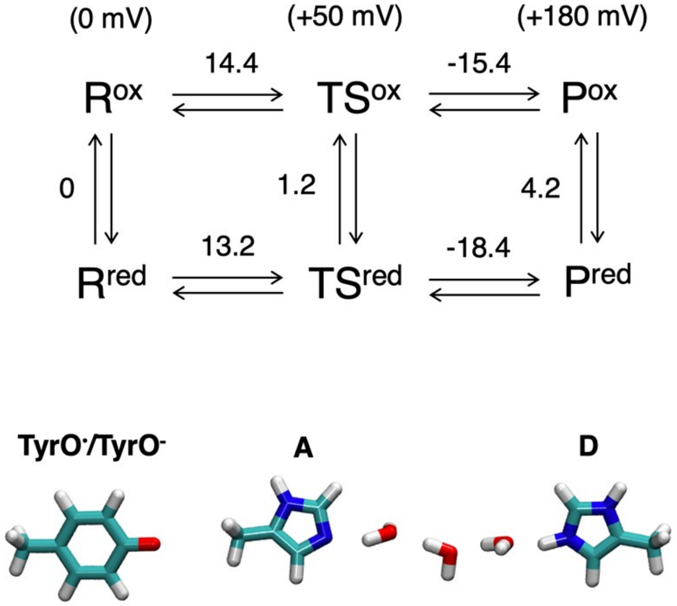

Fig. S4. The pT reaction affects the electron affinities of the redox site, modelled here as a TyrO$^{\bullet}$/TyrO^{-} species (bottom). *Top*: thermodynamic cycle, with energies expressed in kcal mol^{-1}, and relative electron affinities in mV (in parenthesis). The stabilisation of the pT energetics by the reduction reaction (4.2 kcal mol^{-1}), leads to a 180 mV increase in electron affinity of the redox site. *Bottom*: model of the PCET reaction with n = 3 water molecules coupled to the TyrO$^{\bullet}$/TyrO^{-} redox reaction, placed at *ca.* 5 Å of the proton acceptor site. Energies are reported at the B3LYP-D3/def2-TZVP/ε=4 level.

Table S1. Zero-point energy (ZPE) and entropic contributions ($T\Delta S$) at T = 310 for the PCET reaction for the models with n =1, 3, and 5 water molecules, without ($\boldsymbol{E}_f$=0) and with a uniform electric field ($\boldsymbol{E}_f$= 0.05 VÅ^{-1}) applied along the pT direction. TS_M refer to the transition state obtain at the center of the water chain for n=5. Energies are reported at the B3LYP-D3/def2-TZVP level.

			**Δ*ZPE*(kcal mol^{-1})**	***T*Δ*S* (kcal mol^{-1})**	**Δ*E* (kcal mol^{-1})**	**Δ*G* (kcal mol^{-1})**
**E_f = 0**	***n* = 1**	R	0.0	0.0	0.0	0.0
		TS	-4.7	-1.3	14.2	10.9
		P	0.0	0.0	0.0	0.0
	***n* = 3**	R	0.0	0.0	0.0	0.0
		TS	-1.9	-0.7	18.2	17.0
		P	0.3	0.0	-0.1	0.2
	***n* = 5**	R	0.00	0.00	0.0	0.0
		TS_1	-3.4	2.6	16.7	10.7
		TS_M	-3.1	2.6	16.5	10.9
		TS_2	-3.9	0.9	16.7	11.9
		P	0.4	-0.6	0.0	1.0
**E_f=0.05 VÅ^{-1}**	***n* = 1**	R	0.0	0.0	0.0	0.0
		TS	-4.5	-1.3	12.2	9.0
		P	0.1	0.0	-4.0	-4.0
	***n* = 3**	R	0.0	0.0	0.0	0.0
		TS	-1.8	-0.6	14.7	13.4
		P	0.2	-0.1	-7.6	-7.3
	***n* = 5**	R	0.0	0.0	0.0	0.0
		TS_1	-6.4	1.7	12.1	4.0
		TS_M	-5.6	3.5	11.0	1.9
		TS_2	-6.8	2.1	9.6	0.8
		P	0.3	0.0	-11.0	-10.7

References

(1) Becke, A. D. Density-Functional Thermochemistry. III. The Role of Exact Exchange. *J. Chem. Phys.* **1993**, *98* (7), 5648–5652.

(2) Lee, C.; Yang, W.; Parr, R. G. Development of the Colle-Salvetti Correlation-Energy Formula into a Functional of the Electron Density. *Phys. Rev. B* **1988**, *37* (2), 785–789.

(3) Schäfer, A.; Horn, H.; Ahlrichs, R. Fully Optimized Contracted Gaussian Basis Sets for Atoms Li to Kr. *J. Chem. Phys.* **1992**, *97* (4), 2571–2577.

(4) Grimme, S.; Antony, J.; Ehrlich, S.; Krieg, H. A Consistent and Accurate Ab Initio Parametrization of Density Functional Dispersion Correction (DFT-D) for the 94 Elements H-Pu. *J. Chem. Phys.* **2010**, *132* (15), 154104-1–19.

(5) Plessow, P. Reaction Path Optimization without NEB Springs or Interpolation Algorithms. *J. Chem. Theory Comput.* **2013**, *9* (3), 1305–1310.

(6) Huang, J.; Mackerell, A. D. CHARMM36 All-Atom Additive Protein Force Field: Validation Based on Comparison to NMR Data. *J. Comput. Chem.* **2013**, *34* (25), 2135–2145.

(7) Klamt, A.; Schüürmann, G. COSMO: A New Approach to Dielectric Screening in Solvents with Explicit Expressions for the Screening Energy and Its Gradient. *J. Chem. Soc. Perkin Trans. 2* **1993**, No. 5, 799–805.

(8) Ahlrichs, R.; Bär, M.; Häser, M.; Horn, H.; Kölmel, C. Electronic Structure Calculations on Workstation Computers: The Program System Turbomole. *Chem. Phys. Lett.* **1989**, *162* (3), 165–169.

(9) Aoyama, H.; Shimokata, K.; Tsukihara, T.; Yamashita, E.; Katayama, Y.; Ishimura, Y.; Muramoto, K.; Shimada, H.; Yoshikawa, S.; Mochizuki, M.; et al. The Low-Spin Heme of Cytochrome c Oxidase as the Driving Element of the Proton-Pumping Process. *Proc. Natl. Acad. Sci.* **2003**, *100* (26), 15304–15309.

(10) Supekar, S.; Gamiz-Hernandez, A. P.; Kaila, V. R. I. A Protonated Water Cluster as a Transient Proton-Loading Site in Cytochrome c Oxidase. *Angew. Chemie - Int. Ed.* **2016**, *55* (39), 11940–11944.
